# Peat and coconut fiber as biofilters for chromium adsorption from contaminated wastewaters

**DOI:** 10.1007/s11356-015-5285-x

**Published:** 2015-08-29

**Authors:** Kołoczek Henryk, Chwastowski Jarosław, Żukowski Witold

**Affiliations:** Department of Inorganic Technology and Environmental Biotechnology, Cracow University of Technology, Krakow, Poland; Faculty of Chemical Engineering and Technology, Cracow University of Technology, Krakow, Poland

**Keywords:** Canadian peat, Coconut fiber, Cr(III), Cr(VI), Adsorption, Reduction, Kinetics

## Abstract

Batch adsorption experiments were performed for the removal of chromium (III) and chromium (VI) ions from aqueous solutions using Canadian peat and coconut fiber. The Langmuir model was used to describe the adsorption isotherm. The maximum adsorption for peat reached 18.75 mg/g for Cr(III) and 8.02 mg/g for Cr(VI), whereas the value for fiber was slightly higher and reached 19.21 mg/g for Cr(III) and 9.54 mg/g for Cr(VI). Both chromium forms could be easily eluted from the materials. The adsorption of chromium forms to organic matter could be explained in terms of formation of donor-acceptor chemical covalent bound with hydroxyl groups as ligands and chromium as the central atom in the formed complex. The chromate-reducing activities were monitored with the use of electron paramagnetic resonance spectroscopy. The results showed that both adsorption and reduction occurred simultaneously and the maximum adsorption capacity of hexavalent chromium being equal to 95 % for fiber and 92 % for peat was obtained at pH 1.5. The reduction of Cr(VI) in wastewaters began immediately and disappeared after 20 h. Both materials contained yeast and fungi species which can be responsible for reduction of chromium compounds, due to their enzymatic activity (Chwastowski and Koloczek (Acta Biochim Pol 60: 829–834, 2013)). The reduction of Cr(VI) is a two-phase process, the first phase being rapid and based on chemical reaction and the second phase having biological features. After the recovery step, both types of organic materials can be used again for chromium adsorption without any loss in the metal uptake. Both of the materials could be used as biofilters in the wastewater treatment plants.

## Introduction

The discharge of heavy metals in effluents is one of the effects of increasing industrialization that contaminates the environment (Balan et al. 2009; Sollitto et al. 2010; Singh et al. 2004). The environmental pollution by chromate (Cr^6+^, CrO_4_^2−^) is a result of its wide use in industry (tanning, corrosion control, plating, pigment manufacture, and nuclear energy). Cr(VI) is the second most important heavy metal-based pollutant regarded as a priority contaminant by the US EPA (the concentrations of which reach up to 0.18 mM in groundwater and up to 70 mM in soil, according to the US Department of Energy, 2001). The adverse health effects and diverse cellular and molecular reactions make the studies on chromium toxicology and metabolism very crucial in terms of environmental protection and clinical medicine. The toxic action of Cr(VI) arises from its single-electron reduction by flavoenzymes and other redox reactions, i.e., the formation of Cr(V) which initiates the formation of reactive oxygen species (ROS) damaging phospholipids, proteins, and DNA (Stearns and Wetterhahn 1994; Levina and Lay 2004). Chromium compounds are some of the best-documented mutagens and carcinogens among the variety of toxic metal compounds used in industry (Chwastowski and Kołoczek 2013; Yuan et al. 2010). The stable oxidation states of chromium are Cr(VI) and Cr(III) found in different forms depending on pH values (Balan et al. 2009; Mohan and Pittman 2006; Silva et al. 2008). In spite of lower toxicity of Cr(III) compared to Cr(VI), both of the metal forms are harmful. At higher concentrations Cr(III) can also cause adverse health effects because it can coordinate different organic compounds, resulting in inhibition of metallic-enzyme systems (Kotas and Stasicka 2000). The limit for aqueous effluents discharged into waters is about 2 mg/l depending on the country (European Directive 80/778/EC, 1980). Several technologies have been explicated to remove various metals from industrial wastewater, mainly from tannery factories. Although the tannery wastewater contains much more Cr(III) than Cr(VI), under certain circumstances, Cr(III) may be oxidized to Cr(VI), a process that can lead to serious environmental consequences (Gomez and Callao 2006). The corresponding oxidation of Cr(III) to Cr(VI) occurs, particularly in the presence of MnO_2_ and bacteria, but kinetics of the oxidation is slow (Richard and Bourg 1991). For these reasons, simultaneous removal of the oxidized and reduced forms of chromium is of special interest. Many of these processes are complicated, generate wastes, and may be expensive, ineffective, and time consuming. Peat and coconut fiber are inexpensive and widely available natural materials. They consist of different types of organic matter at various stages of decomposition like cellulose, humic and fulvic acids, or lignin. These components contain hydroxyl and weak acidic groups, such as carboxyl and phenolic hydroxide, which can be involved in chemical bonding of metals and polar organics (Balan et al. 2009). The ability of peat to remove various heavy metals like chromium, cadmium, nickel, lead, and copper has been reported by many authors (Brown et al. 2000; da Cerqueira et al. 2012). Typical chromium concentration in tannery wastewater ranges from 170 to 200 μg/g (Milacic and Stupar 1995) which is far lower than the concentration used in this study. High concentration of chromium was used in order to check the maximum possible adsorption on the organic materials. Additionally, the adsorption of both chromium forms and reduction of Cr(VI) in tannery wastewaters (probes taken from the tannery station—southern part of Poland) in the presence of the organic sorbents were observed. The measurements of hexavalent chromium reduction in the wastewaters by coconut fiber and Canadian peat were conducted for the first time. In this study, the sorption capacity of the commercially available Canadian peat and coconut fiber for both chromium (III) and chromium (VI) ions in the concentration equal to 10 mM/L was investigated.

## Materials and methods

Chemicals and reagents used were of high-purity grade. Stock solutions of 10 mM Cr(VI) and 10 mM Cr(III) were prepared using solid K_2_Cr_2_O_7_ and CrCl_3_·6H_2_O then dissolved in demineralized water.

### Preparation of Canadian peat and coconut fiber

In this study, commercially available Canadian peat (bought from the SpillSorb Company) and coconut fiber (bought from the market) were used. Both materials were washed three times with the use of distilled water until the filtrate was clear, then air-dried at room temperature, and sieved through a 100-mesh grid. In order to improve the dissociation of carboxylic groups from both peat and coconut fiber, probe samples of 1 g were treated for 12 h with 10 ml of 0.1 M NaCl solution (Balan et al. 2009). After 12 h, the probes were centrifuged at 3000 rpm for 10 min and the supernatant was discarded. The solid residue was taken for further experiments.

Microbiological tests were performed in order to check whether the material contained microorganisms. Five grams of each material was separately added to two 250-ml flasks. Fifty milliliters of demineralized water was added to the flasks and then the probes were stirred for 3 h at the room temperature. In order to acquire the supernatant, the probes were centrifuged at 3000 rpm for 10 min. The obtained solution was diluted with water and cultured in Petri dish containing 10 ml of growth medium YPD (yeast extract, peptone, dextrose). The composition of YPD was as follows: peptone, 20; yeast extract, 10; saccharose, 20; agarose, 20 (g/l of medium). After the 7 days of incubation, the probes were examined using a microscope. Both of the organic materials contained yeast species, moreover the coconut fiber contained fungi species.

### pH as an influencing factor for removal of Cr(VI) and Cr(III)

Portions of 10 ml of Cr(VI) solution with different pH were transferred into the solid phase extraction columns with 1 g of Canadian peat and 1 g of coconut fiber, respectively. The pH of the solutions were adjusted to the following values: 1.5, 2.5, 3, 4, 5, and 6 using pH-meter and appropriate amounts of 1 M HCl solution. A control sample (pH 4.8) was prepared by adding Cr(VI) without HCl addition. The same steps were taken for Cr(III) solution. Batch adsorption experiments were carried out in 10-ml SPE columns (solid phase extraction) with the same flow rate in each probe (0.1 ml/s).

### Determination of Cr(VI) and Cr(III) in filtered solution

The concentrations of Cr(VI) and Cr(III) were determined in the filtrates. The amount of metal adsorbed on the material was determined as the difference between concentration of stock solution and the concentration of the measured filtrate. Concentration of Cr(VI) was determined with a spectrophotometer at a wavelength equal to 376 nm for Cr(VI) and 601 nm for Cr(III), respectively.

### Determination of Cr(VI) and Cr(III) in wastewater

Wastewater taken from the wastewater treatment plant located in the south of Poland was centrifuged at 5000 rpm/min for 10 min. The supernatant was tested for the presence of Cr(VI) by means of absorption after the reaction with 1,2-diphenylocarbazide at a wavelength of 546 nm. The total amount of chromium was determined using the atomic absorption spectroscopy with a Perkin Elmer spectrophotometer. The concentration of trivalent chromium was calculated as the difference between the concentration of total chromium and hexavalent chromium.

### Electron paramagnetic resonance spectroscopy

In order to investigate reduction processes the experiments were performed at a room temperature using 0.1 g of sorbent and 1 ml of chromium solution. The reduction process was carried out during 120 min with the initial concentration of Cr(VI) equal to 10 mM in each probe. The kinetics of chromate reduction was monitored by means of Cr(V) free radical form determination of spectra using L-band (1.2 GHz) electron paramagnetic resonance (EPR). The spectrometer was equipped with a microwave bridge with the operating frequency of 1.2 GHz and an extended surface coil-type high frequency resonator having an internal diameter of 16 mm. The following settings of the spectrometer were typically used: 30 mW maximum microwave power, 27 kHz field modulation frequency, 40 G magnetic field scan. The scan of the spectra was recorded five times for each measurement and averaged. Every EPR signal was standardized to the free-radical probe (TEMPO) at constant concentration. The spectrum of the probe was taken at the beginning of each measurement. The procedure allows to compare amplitudes of all EPR signals for all the samples.

## Results and discussion

The percentage analyses of peat and coconut organic elements were carried out with Perkin Elmer CHN analyzer type 2400. The measurements were supplemented with an additional analysis to specify moisture and ash content in the samples and carbon, hydrogen, and nitrogen (CHN) composition based on ash and moisture-free state was calculated. Averaged results on the basis of triple repetitions are presented in Table 1. In order to determine chemical characteristics of the organic matter included in Canadian peat and coconut fiber, mass fractions of C, N, and H in ash and moisture-free states were taken into account and mass fractions of protein, carbohydrate, and residual organic content represented as CH_2_ were estimated according to stoichiometric calculations. Table 1 shows the obtained results.

**Table 1:** CHN analysis and estimated chemical composition of both organic materials: peat and coconut fiber

Probe	C (%)	H (%)	N (%)	O (%)	Protein (%)	Carbohydrates (%)	Other inactive organic material (%)
Peat	48.6	5.0	1.3	45.1	8.0	84.6	7.5
	48.7	5.2	1.2	44.9	7.7	84.1	8.1
	48.6	5.1	1.3	45.0	7.8	84.3	7.8
Coconut fiber	46.6	4.5	0.6	48.2	4.0	90.4	5.6
	46.9	4.7	0.6	47.8	4.0	89.6	6.4
	46.8	4.6	0.7	48.0	4.1	90.0	5.9

Results presented at moisture-free state and ash

**Fig. 1:**
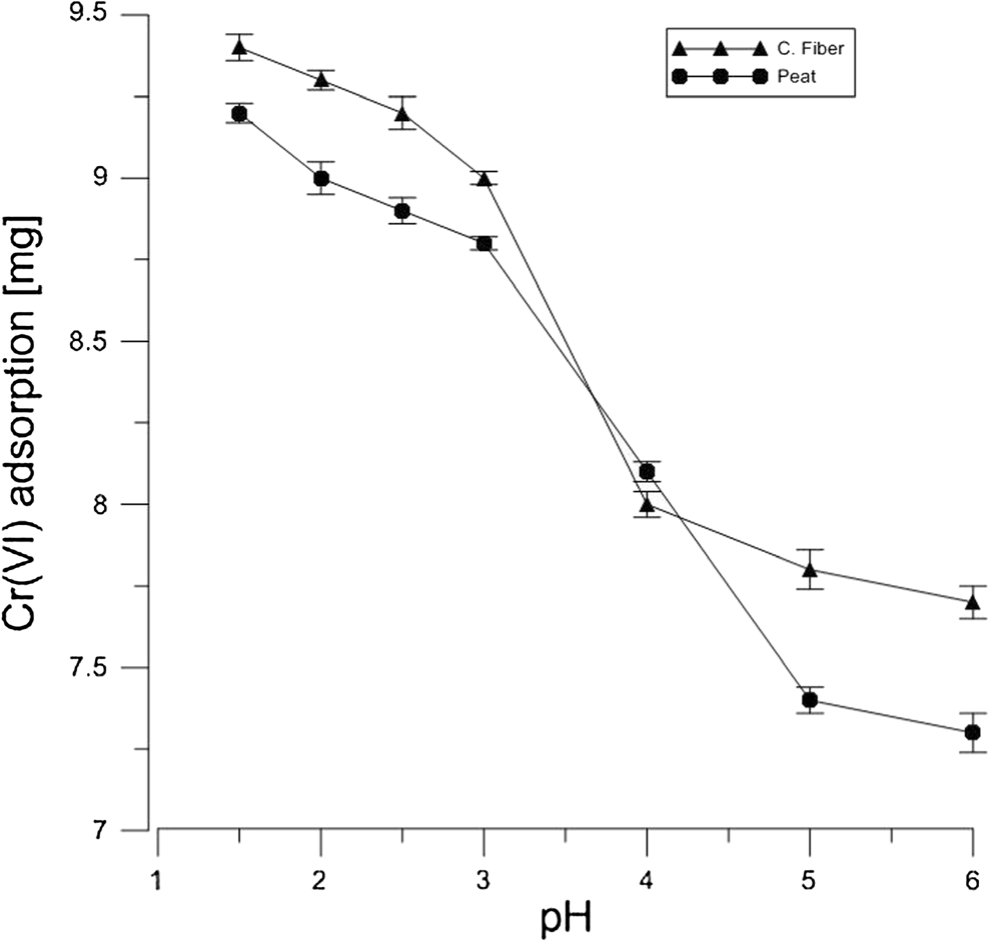
Adsorption of Cr(VI) solution in 20 min by 1 g of peat and coconut fiber as pH function. The concentration of stock solution was equal to 10 mM

**Fig. 2:**
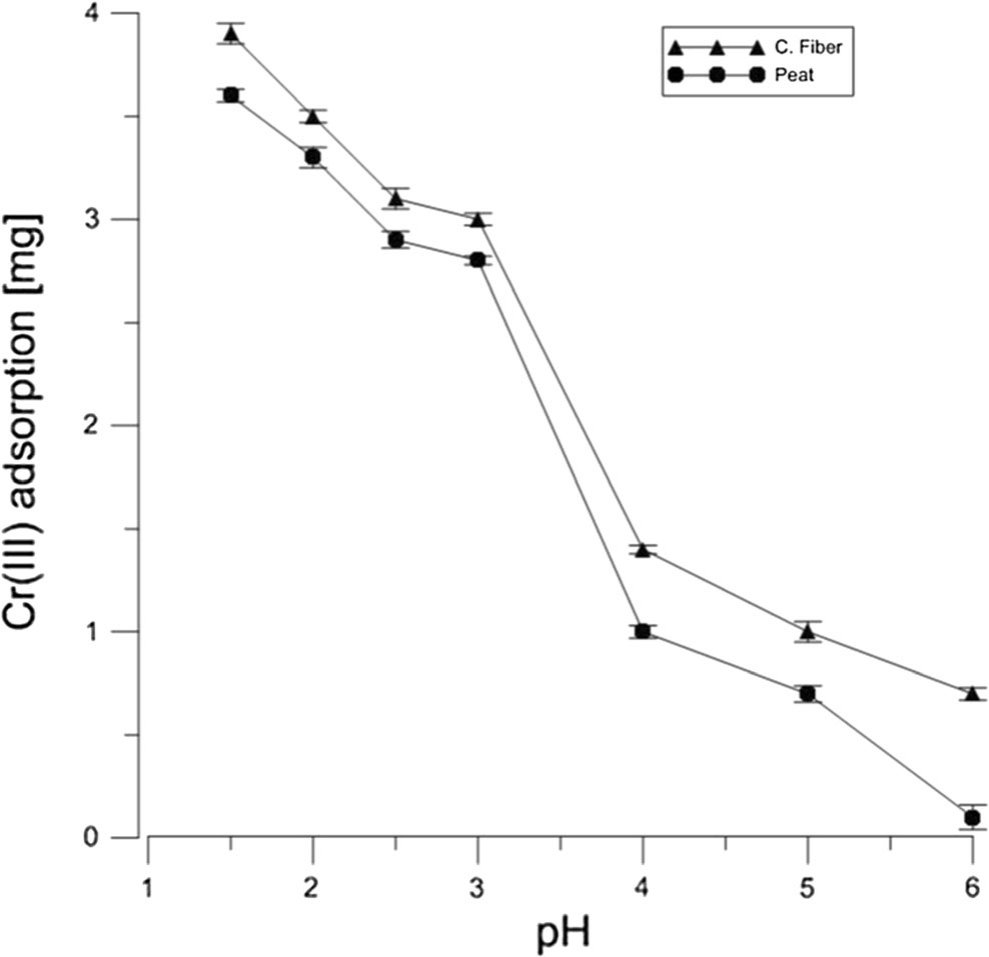
Adsorption of Cr(III) solution in 20 min by 1 g of peat and coconut fiber as pH function. The concentration of stock solution was equal to 10 mM

Figures 1 and 2 show the adsorption process of both hexavalent and trivalent chromium on Canadian peat and coconut fiber at pH values ranging from 2.0 to 6.0. The percentage adsorption of Cr(VI) and Cr(III) in aqueous solution showed high influence of the pH on the adsorption process, e.g., the uptake for peat and coconut fiber at pH 1.5 was equal to 93 and 95 %, respectively. Similar tendency can be observed for the Cr(III). Organic content is higher by about 5 % in fiber compared to peat. The similar results were seen in the case of carbohydrates. The different concentration of the groups in each material can be responsible for the adsorption difference between peat and coconut fiber as presented in Figs. 1 and 2.

**Scheme 1:**
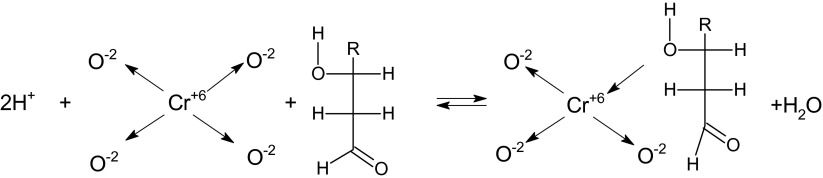
Mechanism of the Cr^+6^ ions interaction with hydroxyl groups of organic material in acid conditions

The proposed explanation of interactions between the hydroxyl groups and the central ion of the chromium complexes can be related to the electronic structure of the chromate anion according to the reaction (Scheme 1):

After elimination of the oxygen atom, the free orbital of chromium is able to accept an unbound electron pair. It can originate from the oxygen atom of the hydroxyl group of an organic sorbent. As a result of the interaction, chromium sorption occurs and the donor-acceptor reaction (1) is strongly related to pH due to the Le Chatelier’s principle.

Such a donor-acceptor reaction induces an additional interaction between chromium complex oxygen ion and other carbonyl or hydroxyl groups of the sorbent, which results in the reduction of central chromium ion as chemical consequences.

The adsorption process of Cr(VI) followed by its reduction to Cr(III) was also detected on magnetite nanoparticles (Yuan et al. 2009).

A more complicated mechanism can be noticed for chromium (III) cation, where series of chemical equilibria are related.

On the one hand, hydrolysis of the cation leads to various kinds of complexes [Cr(OH)_*n*_(H_2_O)_*m*_]^+3 − *n*^, where *n* + *m* = 6 at higher pH. On the other hand, at low pH, a dominant form of chromium with *n* = 0, *m* = 6 is observed and all of the aqua-ligands are weakly bound to Cr. In this situation, free electron pairs from organic material hydroxylic groups can compete with water molecules more effectively than with hydroxide anion and can chelate the central Cr ion. As the result, the adsorption which depends on the pH value occurs again. The high pH values, *n* = 2 and *m* = 4, make this form of complex dominant due to competition between OH^−^ and water molecules. At high pH, the equilibrium reaction is shifted to the complex of Cr and OH^−^. It results in the fact that the adsorption of the Cr^+3^ onto the organic material is negligible.

The above-mentioned explanation constitutes only one aspect of the adsorption mechanisms. The other, in which carboxylic and amine groups are involved, is discussed below.

**Fig. 3:**
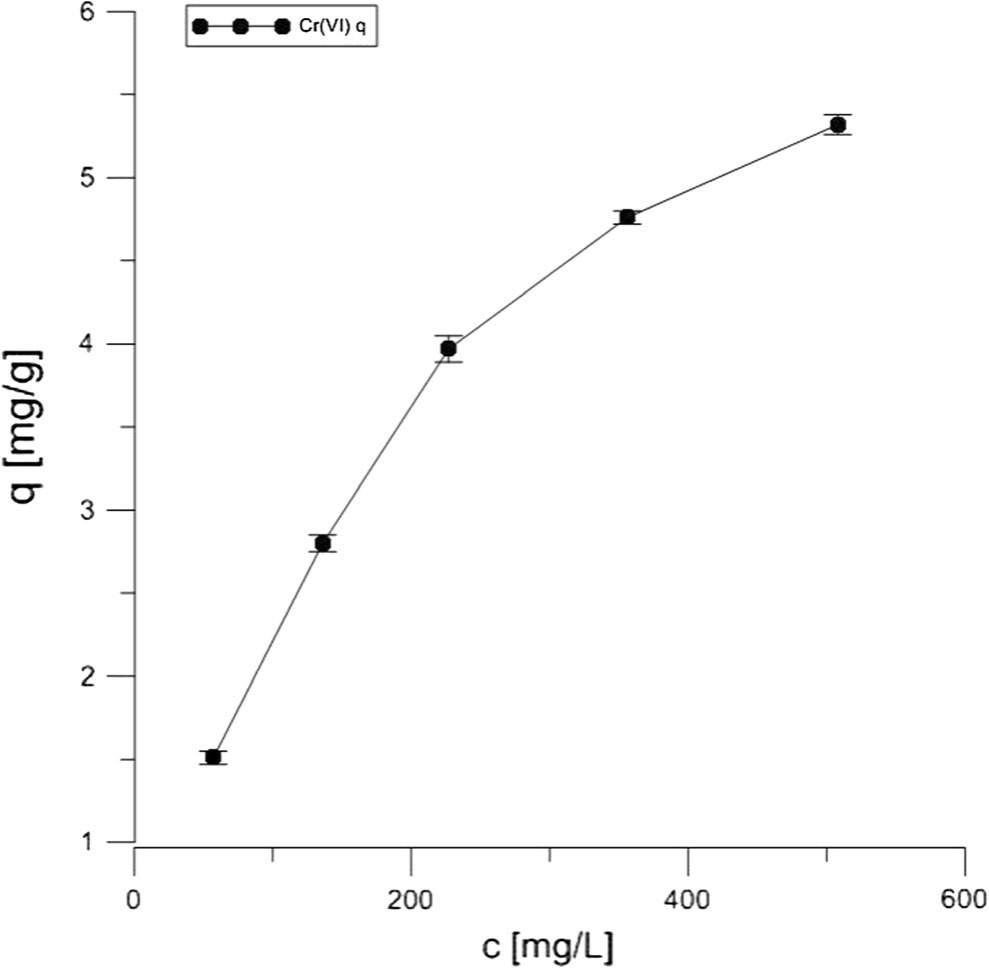
Langmuir isotherm of 10 mM Cr(VI) onto peat during 20 h of contact

**Fig. 4:**
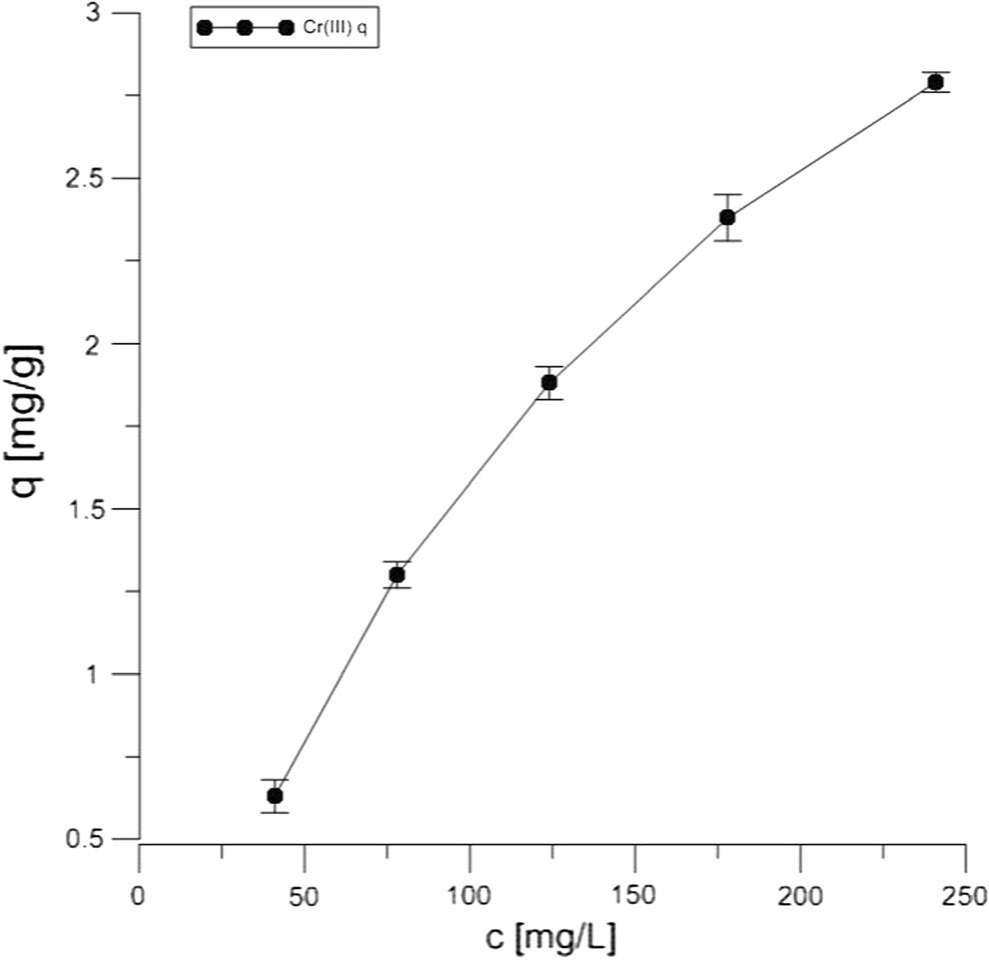
Langmuir isotherm of 10 mM Cr(III) onto peat during 20 h of contact

**Fig. 5:**
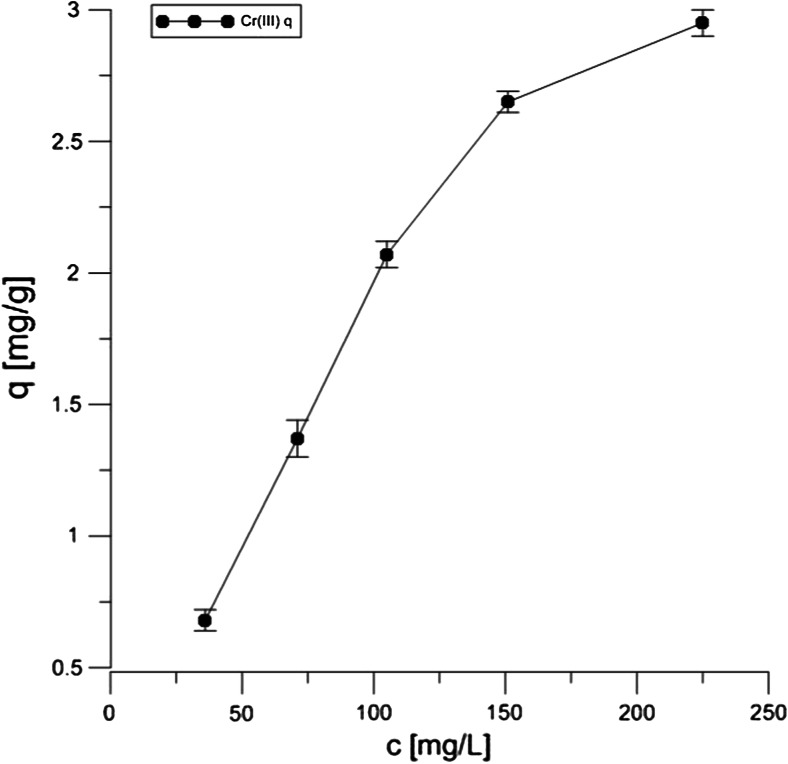
Langmuir isotherm of 10 mM Cr(III) onto peat with NaCl during 20 h of contact

According to the literature data, the adsorption of both Cr forms on the peat and coconut fibers is effective at as low pH as 1.5 (da Cerqueira et al. 2012; Nakayasu et al. 1999). However, such low pH is not preferred as far as the wastewater treatment process is concerned because it requires the use of large amounts of reagents necessary to obtain a low pH and pH-adjusting reagents to the neutral range at the final stage of the effluent treatment. Therefore, the data presented in Table 2 describe chemical parameters of the adsorption process at pH 4.5. The Langmuir sorption isotherms of both chromium III and VI on Canadian peat (and NaCl-treated form) are shown in Figs. 3, 4, and 5. The experiments were done in the presence of 100 mM NaCl, in which case stronger adsorption was observed in comparison with tests carried out without the use of the salt (data not shown). It was assumed that the distribution of chromium ions was described by sorption isotherms which illustrated the distribution of adsorbate species between the solid phase and the liquid phase in equilibrium state.

**Table 2:** Parameters for the sorption of Cr(VI) and (III) on Canadian peat

Isotherm parameter		Peat + Cr(VI) (pH = 4.5)	Peat + Cr(III) (pH = 4.5)	Peat + NaCl + Cr(III) (pH = 4.5)
Langmuir	*q*_0_ (mg/g)	8.02	16.72	18.75
	*K*_*L*_ (l/mg)	0.004	0.0009	0.001
	*R* ^2^	0.997	0.998	0.998

The Langmuir isotherm adsorption model is based on the assumption that maximum adsorption corresponds to a monolayer of solute species on the sorbent surface, containing a finite number of energetically equivalent sites. The equation can be used in the following linearized form:

$$ \frac{1}{q}=\frac{1}{q_0{K}_L{L}_C}+\frac{1}{q_0} $$

where

*q*: the amount of solute adsorbed on the material at equilibrium (mg/g)
*q*_0_: maximum amount of solute adsorbed (mg/g)
*K*_*L*_: constant related to the binding energy of the solute (l/mg)
*L*_*C*_: concentration of Cr in solution after equilibrium

The parameters of Langmuir isotherm were evaluated from the slope and the intercept of the corresponding plots and they are summarized in Table 2.

The *R*^2^ values from Table 2 show that the chromium ion sorption is well characterized by the Langmuir model, indicating the formation of a monolayer coverage of the adsorbate at the outer surface onto the peat and coconut fiber, respectively. The highest adsorption capacity was obtained for the Canadian peat treated with NaCl in comparison with the untreated material, as indicated by the *q*_0._ Very similar data were observed in case of coconut fiber and NaCl (data not shown). A relatively low value of sorption equilibrium constant *K*_*L*_ suggests a weak interaction for both chromium forms with peat sorbent. However, Cr(VI) is more strongly bound on the adsorbent surface than the Cr(III) ion, probably additional chemical groups are involved in the adsorption processes, e.g., carboxylic and amine groups, thus, the *q*_0_ indicates more active sites responsible for the binding. The observation is in accordance with the data of Kyziol et al. (2006), where the authors showed that not all carboxylic groups were involved in Cr(III) binding.

In the case of Cr(III) at pH 4.5 ions existed as hydrocations (Cr(H_2_O)_6_^+3^ or Cr(OH)(H_2_O)_5_^+2^), the constant of the first degree of hydrolysis is equal to 2.0 × 10^−4^ in 25 °C (Hiroishi et al. 1998) which means that at pH 3.7, the solution might be an equimolar mixture of Cr(H_2_O)_6_^+3^ or Cr(OH)(H_2_O)_5_^+2^ while Cr(H_2_O)_6_^+3^ could dominate only at lower pH. The constant of the second stage of hydrolysis is equal to 2 × 10^−6^, which means that at pH 5.8 there is an equimolar mixture of Cr(OH)(H_2_O)_5_^+^ and Cr(OH)_2_(H_2_O)_4_^+1^ at the much lower concentration of Cr(H_2_O)_6_^+3^ in comparison with the two above. Thus, at the range of 3.7–5.8, the Cr(OH)(H_2_O)_5_^+2^ is a dominant form with the possibility of moving the equilibrium towards the Cr(H_2_O)_6_^+3^ in the case of the occurrence of a factor (in our case the sorbent) shifting the balance in accordance with the contradiction rule and the diagram shown above. This shift and sorption as its consequence is the greater the more the pH is near the 3.7 value.

**Scheme 2:**
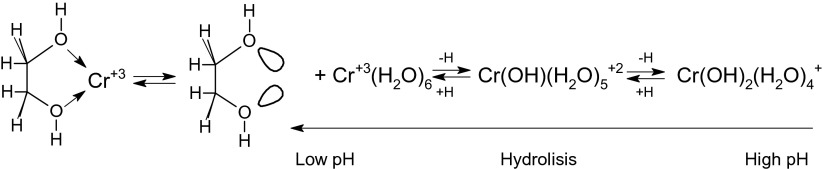
Mechanism of the Cr^+3^ ions interaction with the organic material

At this pH, Cr(III) ions are bound to hydroxylic groups of peat as it was suggested in Scheme 2. In the experimental conditions, the strength of such a binding and the treatment of peat with NaCl result in a higher number of hydroxylic and carboxylic groups available for the interaction. Then the change of *q*_0_ is observed. The bigger number of Cr(III) than Cr(VI) ions bound on the peat can suggest that the negatively charged groups of adsorbent, responsible for the Cr(III) binding, are exposed and divided into at least two classes. Cr(VI) anion is more strongly bound on the sites of “first class,” e.g., amine-protonated groups, since the smaller number of the ions are bound. The opposite situation is observed in the case of Cr(III) which is positively charged—the higher number of ions are bound with sites of “second class” fully exposed to the surface, e.g., hydroxyl groups.

In the work of Cerqueira et al. (2012), the constant for Cr(III) was significantly higher than the values presented in Table 2. It indicates the strong interaction of chromium with the subtropical peat which the authors used in their experiments. As it is known, the organic composition of the peat depends on the environmental conditions in which the peat was formed. The source of such a discrepancy can be connected with the origin of the botanical formation of peat deposits, e.g., in this work, the peat was obtained from an arctic area.

**Fig. 6:**
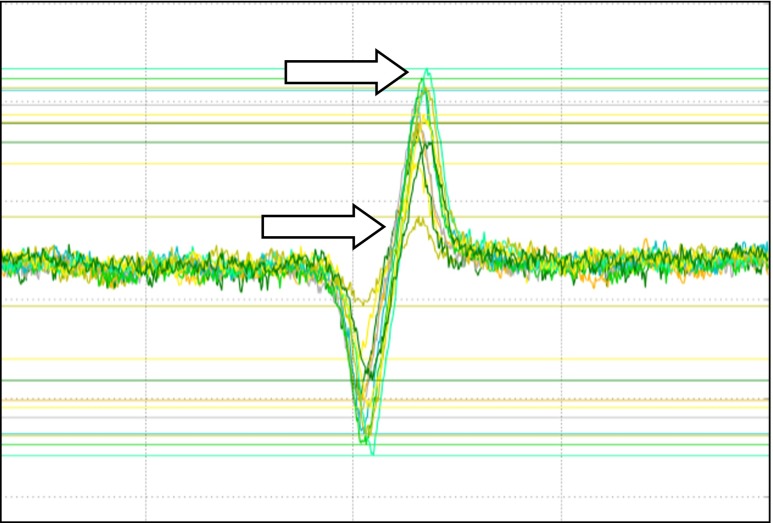
Reduction of Cr(VI) from wastewater by peat (pH = 4.6)measured with EPR L-band spectra of Cr(V). The *upper arrow* indicates the maximum reduction of Cr(VI) in the middle of the experiment. The *lower arrow* indicates the minimum reduction of Cr(VI) at the end of the reduction process. The time of the reduction process was equal to 180 min

Figure 5 illustrates the reduction process of tannery wastewater containing 0.458 ± 0.002 mg/l of the total chromium. The signal coming from the Cr(V) ion suggests that the Cr(VI) is also present in the examined probe and its concentration reaches about 30 % of the total chromium. The experiments carried out with the use of EPR L-band showed that the reduction of Cr(VI) starts in the first seconds after the addition of wastewater to the organic material. After 20 h of the reduction process, there were no signs of reduction which suggests that all of the hexavalent chromium was reduced to the trivalent chromium (Fig. 6).

**Fig. 7:**
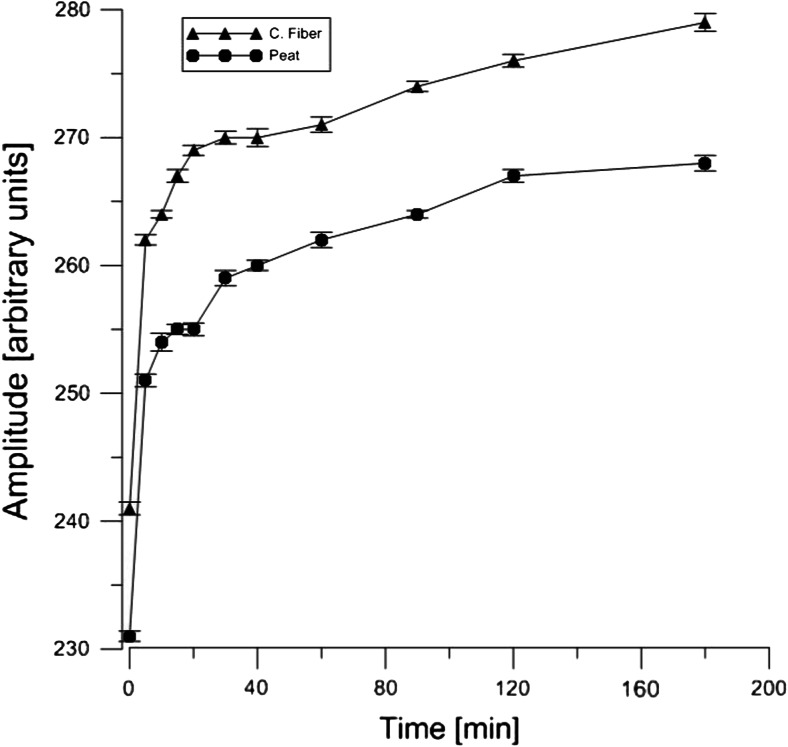
Comparison of the reduction process of 10 mM Cr(VI) by the Canadian peat and coconut fiber

In the case of both organic materials treated with the wastewater from the treatment plant, the reaction of Cr(V) formation starts right after the addition of waste to the organic materials. As can be seen in Fig. 7, there are two phases of the reduction process. The first phase is very rapid and starts right after the contact between organic materials and wastewater containing Cr(VI). The second phase starts after about 18 min and it is slower. After 160 min of the reduction measurement, the process was still ongoing. The complete reduction was observed after 20 h of the incubation process (data not shown). The two-phase reduction process can be explained in terms of chemical reduction (first phase) and activity of bacterial-yeast microorganisms which can reduce the Cr(VI) enzymatically. The similar phenomena were observed in the yeast reduction case.

As suggested by Sharma D.C. and Forster C.F. (1995) the chemical reduction of Cr(VI) is caused by the oxidation of peat particles and arising protons are responsible for the reduction: HCrO_4_^−^ + 7H^+^ + 3e^−^ Cr^3+^ + 4H_2_O

The slight differences between coconut fiber and Canadian peat could be connected with the differences in the presence of the hydroxyquinone/quinine redox couple which increases the reduction process at lower pH due to lowering of the redox potential of the –HCrO_4_ species, which is predominant under acidic conditions (da Cerqueira et al. 2012)

## Desorption studies

In order to check the possibility of reusing both of the organic materials (Canadian peat, coconut fiber), several elutions with demineralized water were made. Portions of 10 ml of demineralized water were added to the SPE columns containing materials with adsorbed chromium ions. After each rinse, the filtrate was tested for the presence of chromium compounds using the above-mentioned methods. After the third washing, there were no signs of Cr in the filtrate. The sum of the chromium content in the filtrates was roughly equal to the amount of chromium applied to the column. The results indicate that both chromium (VI) and (III) ions can be easily eluted. This fact is in agreement with the low interaction between ions and the binding sites on the organic material surface. After the recovery, both organic materials can be used again for chromium adsorption without any loss in the metal uptake. The obtained results show that both materials can be used as biofilters in the wastewater treatment plants for removal of Cr(III) and Cr(VI) ions.

## Conclusions

Both Canadian peat and coconut fiber were able to remove Cr(VI) and Cr(III) from aqueous solution in simultaneous processes of reduction and adsorption onto the material. The coconut fiber was slightly better in terms of adsorption of both Cr(VI) and Cr(III) and reduction of Cr(VI). The adsorption of chromium (VI) could be mainly explained by the fact that there was an interaction between the hydroxyl groups of carbohydrates present in the studied organic matters and central chromium ion. The carboxylic and amine groups of organic matter were also involved with regard to adsorption in the case of cationic (III) forms of the metal. The reduction of Cr(VI) is a two-phase process. The first phase is a rapid chemical reaction, whereas the second phase is longer and probably based on enzymatic reactions.

The results show that both materials can be used as biofilters’ bed in the wastewater treatment plants for removal of both Cr(III) and Cr(VI) ions as well as reduction of the latter form of the metal. Although adsorption reaction is very effective at low pH as can be seen in Figs. 1 and 2, the process of decreasing the pH value to 1.5 is expensive. It seems that the appropriate corrections of pH values are needed to establish the sorption and desorption states of an organic solid bed, e.g., pH 4.5 is equivalent to 0.03 mmol/dm^3^ H_3_O^+^ concentration, and therefore, a moderate amount of reagents allows pH change from 7 to 4.5, necessary for the purification process. The proposed models of chemical sorption/desorption states make it possible to select the materials that can be used in the adsorption and reduction processes, the selection being based on the chemical structure of a sorbent.
